# Forensic Psychiatry—End of Year Reflection

**DOI:** 10.3389/fpsyt.2018.00714

**Published:** 2018-12-18

**Authors:** Thomas Nilsson

**Affiliations:** Department of Forensic Psychiatry, Institute of Neuroscience and Physiology, University of Gothenburg, Gothenburg, Sweden

**Keywords:** forensic, psychiatry, reflection, recommendation, research topics

While we now are approaching the end of 2018, the time has come to lock back and summarize some of the highlights and achievements during the time that has passed. First of all, let me state that I am very pleased to see that the section of Forensic Psychiatry has increased in almost every aspect. A number of Associate Editors as well as Review Editors have joined our team during the last 18 months, clearly broadening our field of expertise. There has also been a noticeable rise in number of manuscript submissions during this period, covering a broad array of topics. This is promising, laying the ground for the section Forensic Psychiatry to become a leading open access journal dedicated to questions within the field of forensic psychiatry. High ambitions like this will make demands on all of us, but I am so far very pleased with the engagement shown by you all. Therefore, let me also state that I appreciate your good work and that I am confident that *Frontiers in Psychiatry*, Forensic Psychiatry, the coming year will improve its contribution to the practice and understanding of forensic psychiatry.

Among the manuscripts published, a couple stands out as especially informative and interesting, also evidenced by a high number of views and downloads. One of these that I could recommend is “Persistency of Cannabis Use Predicts Violence Following Acute Psychiatric Discharge” (1), suggesting an association between cannabis use and violence in psychiatric patients. This information is highly relevant in our times when calls for legalization are heard in several western countries. Another paper worth reading is “eHealth in Treatment of Offenders in Forensic Mental Health: A Review of the Current State” (2). eHealth as a method for treatment of psychiatric problems has emerged as a promising way to use modern technologies and tailor interventions to individual needs. In this paper experiences of the use of this method within forensic psychiatry are summarized and future possibilities and developmental ways are discussed. An additional paper worth reading is “Mapping Systematic Reviews on Forensic Psychiatric Care: A Systematic Review Identifying Knowledge Gaps” (3), since it gives an overview of knowledge gaps within our field. The lack of evidence is especially serious with regard to treatment interventions of mentally disordered offenders who in many cases receives involuntary care for several years, thus calling for the need of research and knowledge accumulation.

A couple of Research Topics have also been launched during the last 2 years. One that has generated interesting papers is “Caring for Those Who are Neglected and Forgotten: Psychiatry in Prison Environments^1^.” Here many aspects concerning mental health among prisoners and their need of support and treatment have been in focus for submitted papers. I can really recommend interested readers to take part of them, as they give voice to the needs of a disregarded group with an increased risk of mental illness, poor health outcome and suicidality. An ongoing topic is “Compulsory Interventions in Psychiatry: an Overview on the Current Situation and Recommendations for Prevention and Adequate Use^2^,” which has attracted many authors. Hence, this topic will generate knowledge about compulsory interventions, their effects, and their consequences for patients in the upcoming year. The research topic “What Works for Forensic Psychiatric Patients: From Treatment Evaluations to Short and Long-Term Outcomes^3^” has just been dispatched. Here contributors are invited to present studies of interventions of different kinds for mentally disordered offenders. Thus, I am convinced that Frontiers in Psychiatry, section Forensic Psychiatry, in the nearby future will be able to publish papers highly relevant to our field, contributing to the development of knowledge about such important questions as how to best meet the needs of forensic psychiatric patients and give them treatment and support that have the opportunity to make a difference.

## Author Contributions

The author confirms being the sole contributor of this work and has approved it for publication.

### Conflict of Interest Statement

The author declares that the research was conducted in the absence of any commercial or financial relationships that could be construed as a potential conflict of interest.

## Footnotes

^1^https://www.frontiersin.org/research-topics/6913/caring-for-those-who-are-neglected-and-forgotten-psychiatry-in-prison-environments#overview

^2^https://www.frontiersin.org/research-topics/8446/compulsory-interventions-in-psychiatry-an-overview-on-the-current-situation-and-recommendations-for

^3^https://www.frontiersin.org/research-topics/9303/what-works-for-forensic-psychiatric-patients-from-treatment-evaluations-to-short-and-long-term-outco
